# Case report: Clinical efficacy of deep brain stimulation contacts corresponds to local field potential signals in a patient with obsessive-compulsive disorder

**DOI:** 10.3389/fpsyt.2023.1279972

**Published:** 2023-11-23

**Authors:** Korrina A. Duffy, Elizabeth A. Fenstermacher, John A. Thompson, Jody Tanabe, Moksha S. Patel, Steven Ojemann, Rachel A. Davis

**Affiliations:** ^1^Department of Psychiatry, University of Colorado School of Medicine, Anschutz Medical Campus, Aurora, CO, United States; ^2^Department of Neurosurgery, University of Colorado School of Medicine, Anschutz Medical Campus, Aurora, CO, United States; ^3^Department of Neurology, University of Colorado School of Medicine, Anschutz Medical Campus, Aurora, CO, United States; ^4^Department of Radiology, University of Colorado School of Medicine, Anschutz Medical Campus, Aurora, CO, United States; ^5^Division of Hospital Medicine, University of Colorado School of Medicine, Anschutz Medical Campus, Aurora, CO, United States

**Keywords:** OCD, DBS, neuromodulation, brain sensing, ventral striatum, BNST, biomarker, psychiatric neurosurgery

## Abstract

**Introduction:**

Deep brain stimulation (DBS) is often effective in treating severe obsessive-compulsive disorder (OCD) when traditional therapeutic approaches have failed. However, optimizing DBS programming is a time-consuming process. Recent research in movement disorders suggests that local field potentials can dramatically speed up the process of identifying the optimal contacts for stimulation, but this has not yet been tested in a patient with OCD.

**Methods:**

In a patient with severe OCD, we first determined the optimal contact for stimulation for each hemisphere using traditional monopolar and bipolar review and then tested whether the clinically optimal contact in each hemisphere corresponded to local field potential signals.

**Results:**

Overall, we found that clinical efficacy corresponded with the contacts that showed the strongest local field potential signals across multiple frequency bands.

**Discussion:**

Our findings are the first indication that local field potentials could guide contact selection in patients with OCD. If validated in a larger sample, this methodology could decrease time to clinical benefit and improve accuracy in patients that are difficult to assess using traditional methods. Further research is needed to determine whether local field potentials could be used to guide finer resolution in programming parameters.

## 1 Introduction

The goal of deep brain stimulation (DBS) programming is to identify parameters that provide maximal clinical benefit while minimizing adverse effects. Optimizing DBS programming for psychiatric conditions, such as obsessive-compulsive disorder (OCD), is a complex and time-consuming process and relies on assessing real-time changes in proxies (improvements in mood, anxiety, and energy) thought to predict later reduction in OCD symptoms. Recent research in movement disorders suggests that use of local field potentials (LFPs) could increase the efficiency of identifying the optimal contact for stimulation. Although most of these studies have been conducted in patients with Parkinson’s disease (PD) (1–10), two recent case reports have demonstrated that LFPs correspond to the most clinically efficacious contact in patients with either epilepsy, dystonia, or seizures (11, 12). Given the delayed onset of improvement with DBS stimulation in OCD (compared to immediate response in PD and essential tremor), using LFPs in patients with OCD to guide contact selection has the potential to improve programming accuracy and reduce time to clinical benefit (12, 13). In a patient with OCD, we tested whether LFP spectral power corresponded with the most clinically efficacious contacts selected using current standard of care monopolar and bipolar reviews conducted by a clinician.

## 2 Methods

**Figure 1:**
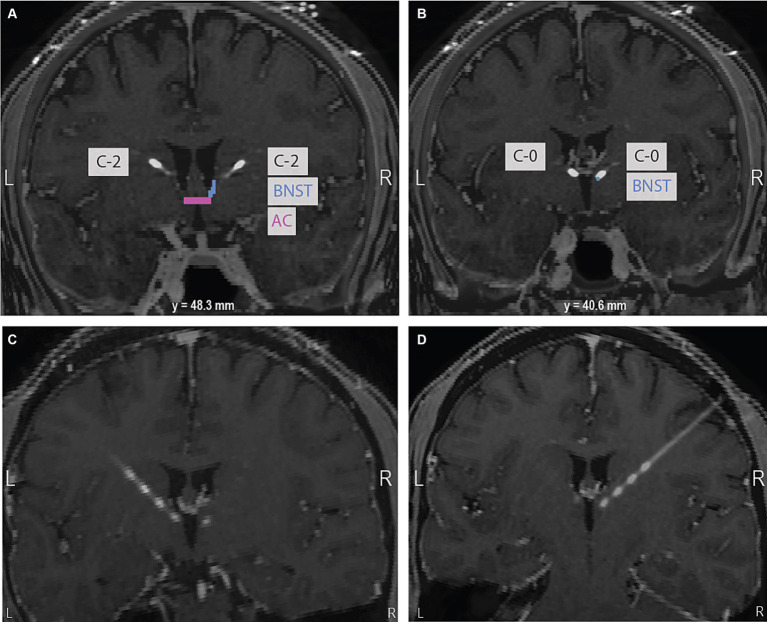
**(A)** At the level of the anterior commissure (AC) highlighted in pink, contact 2 in the right hemisphere is shown in the anterior limb of the internal capsule (ALIC). The bed nucleus of the stria terminalis (BNST), the intended target, is highlighted in blue. **(B)** 40.6 mm posterior to the AC, contact 0 in the right hemisphere can be seen in the expected region of the BNST. The bottom panel of oblique coronal images shows contacts 0 to 3 on the left **(C)** and right **(D)**. Images are in neurological convention.

The patient is a 31-year-old male physician with intractable OCD that severely impaired his functioning and a significant family history of OCD with diagnosed disease in both maternal and paternal cousins. He first experienced OCD symptomatology when he was 4 to 5 years old and developed severe and persistent symptoms towards the end of high school. He first entered exposure response prevention at age 18 and reported working with 12 clinicians for medication management and therapy before presenting to our clinic in September 2019. At that time, his OCD diagnosis was confirmed with structured clinical interviewing. His preoperative average Yale-Brown Obsessive-Compulsive Scale (YBOCS) score was 35, indicative of extreme OCD. His obsessions centered around contamination fears, with a significant focus on a fear of fecal incontinence and fecal contamination. The patient consented to surgical implantation of bilateral DBS leads in the ventral capsule/ventral striatum (VC/VS) with the most ventral contact (contact 0) targeting the bed nucleus of the stria terminalis (BNST) and the more dorsal contacts targeting fibers running through the anterior limb of the internal capsule (ALIC) identified by diffusion tensor imaging tractography (University of Colorado COMIRB 14–0554; FDA Humanitarian Device Exemption H050003). See Figure 1 for a brain scan depicting implanted electrodes and Supplementary Figure S1 for preoperative diffusion tensor imaging tractography.

In September 2021, surgery occurred under general anesthesia, and bilateral DBS leads (Model 3,391, Medtronic, Minneapolis, MN, United States) were placed using MRI guidance. Bilateral implantable pulse generators (IPGs) (Percept B35200) were placed 2 weeks after the initial surgery and stimulation was turned on 1 week later. See Supplemental materials for details on his medications prior to surgery and after stimulation was turned on.

In October 2021, optimal contacts were selected using monopolar and bipolar reviews, conducted during separate sessions, based on a previously described programming protocol (14). Clinical response was based on patient reported mood, anxiety, and energy as well as clinician observed smiles, laughs, and overall engagement (15). In November 2021, his optimal treatment stimulation parameters were determined to be left case +, contact 0 negative, 4.5 mA, 100 μsec, 60 Hz; right case +, contact 0 negative, 4.5 mA, 90 μsec, 135 Hz. See Supplemental materials for details on why split stimulation frequencies were used.

The patient had a good clinical response to DBS. Before surgery, his YBOCS score was 35 and his Montgomery-Asberg Depression Rating Scale (MADRS) score was 17. Nine days after stimulation was turned on, his YBOCS score was 20 and his MADRS score was 7, indicating a rapid reduction in OCD and depression symptoms. A month after his optimal treatment stimulation parameters were set, he had a YBOCS of 16 and MADRS of 6.

**Figure 2:**
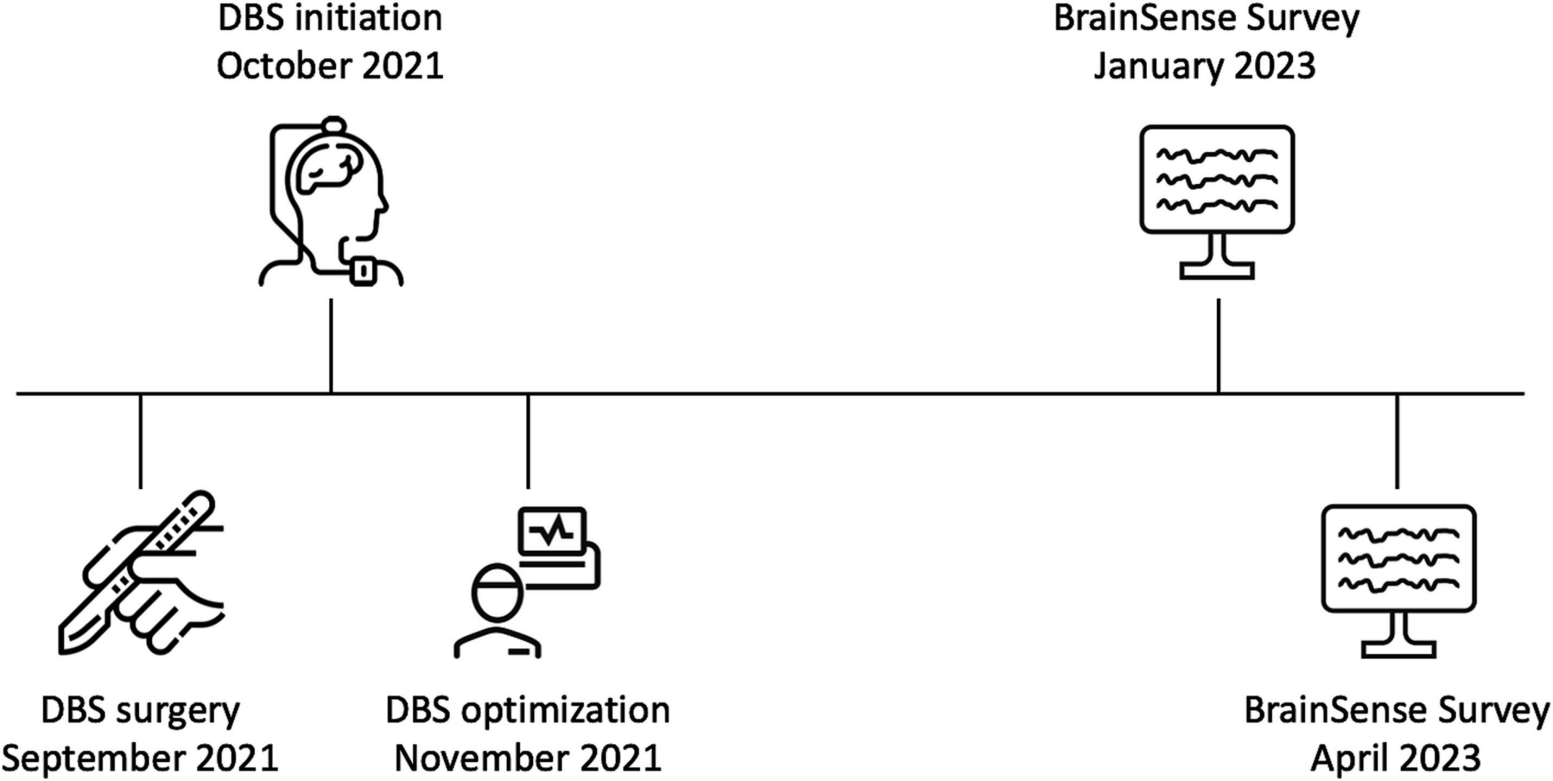
Timeline of key study events.

We used BrainSense Survey (BSS) to test whether the contacts in the left and right hemispheres with the strongest relative LFP signal would align with the contacts identified using monopolar and bipolar review. The first BSS recording session occurred in January 2023 and the second BSS recording session occurred in April 2023. We did two separate sessions so we could test whether our findings replicated over time. The patient consented to having LFPs recorded between all six contact combinations (i.e., 0–3, 0–2, 1–3, 0–1, 1–2, 2–3) for each hemisphere using the BSS feature while stimulation was turned off. We assessed the strength of the signal from each contact pair to identify the contact with the greatest signal in each hemisphere and compared these results to findings using traditional monopolar and bipolar surveys. Recordings were done for approximately 20s per contact pair at a sampling rate of 250 Hz. The spectral output contained bin sizes of 0.98 Hz. Because recordings could only be conducted simultaneously with distinct contact pairs with no shared contacts (e.g., 0–1 and 2–3), some contact pairs were recorded simultaneously while others were recorded consecutively over the recording period for each hemisphere. LFPs were recorded only once at baseline for each BSS session. For a timeline of key study events, see Figure 2.

## 3 Results

Because contacts that are farther apart will generally have greater power differentials, it is essential to compare the power differentials of contact pairs that are the same distance apart from one another (e.g., 0–2 and 1–3) to determine which contact pair contains the contact with the strongest signal. Taking all comparisons into account, it is possible to hone in on the contact from which the strongest LFP signal is originating.

**Figure 3:**
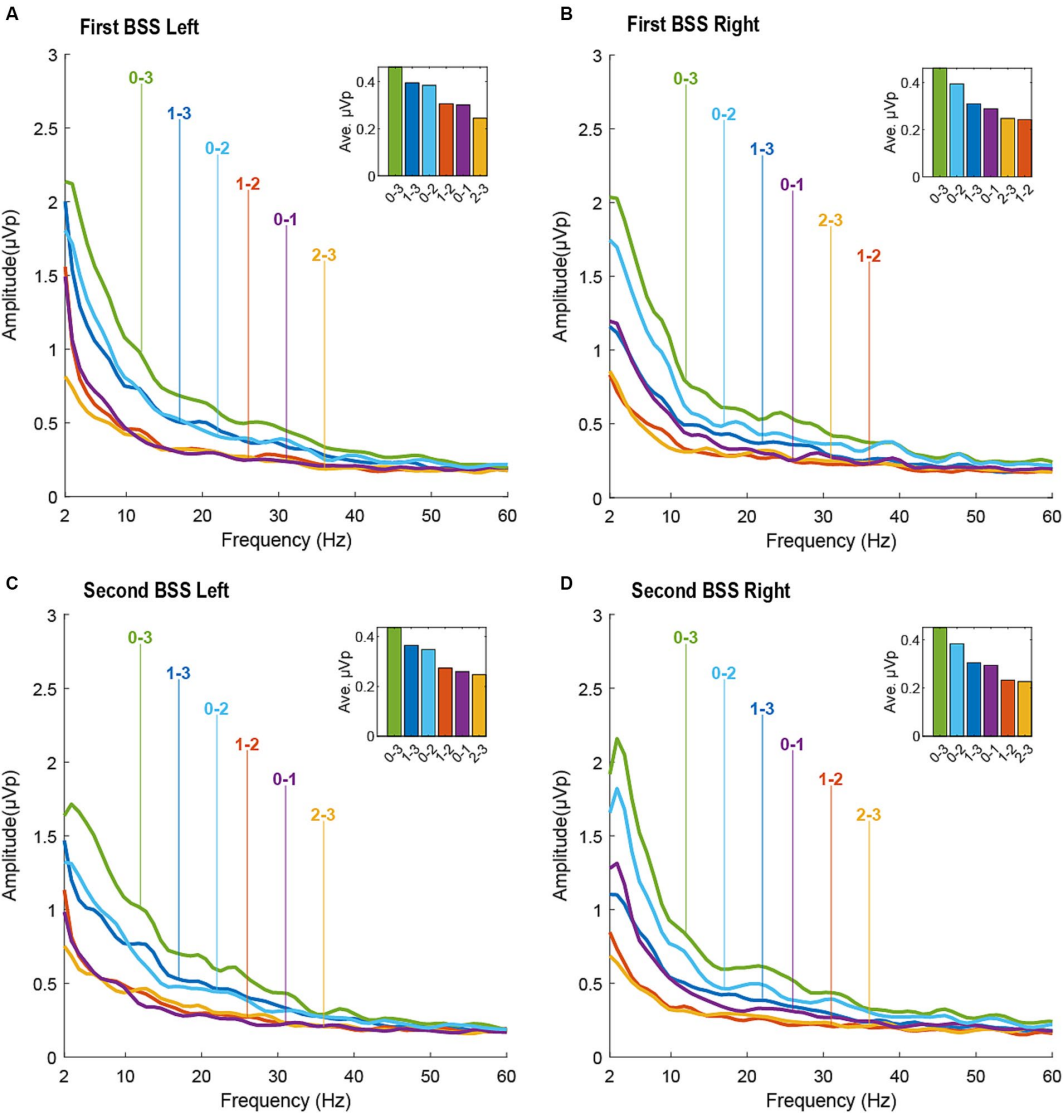
Local field potentials (LFPs) measured during the first BrainSense Survey (BSS) session (top panel: **(A)**. left hemisphere; **(B)**. right hemisphere) and second BSS session (bottom panel: **(C)**. left hemisphere; **(D)**. right hemisphere). LFPs are presented for all differentials between contacts 0–3.

On the right lead, for both BSS sessions, the contact pair that was the farthest apart (i.e., 0–3) had the highest power differential across all frequency bands. For contact pairs that were the next farthest apart, 0–2 showed a greater power differential than 1–3 across all frequency bands. For contact pairs that were the closest together, 0–1 had a greater power differential than 1–2 and 2–3 across all frequency bands. Given the commonality of contact 0 across these comparisons, these findings suggests that the strongest LFP signal is in closest proximity to contact 0. In the theta range, we detected a theta peak that seemed to originate most strongly from contact 0. Contact 0 was determined to be the contact with the greatest clinical benefit based on monopolar and bipolar review, indicating that the contact with the strongest signal corresponds to the contact with the greatest clinical benefit in the right hemisphere, see Figure 3; Table 1.

**Table 1:** Power differentials for each contact pair within each power band for the left and right leads.

Left lead							Right lead					
Power band	0–3	0–2	1–3	0–1	1–2	2–3	0–3	0–2	1–3	0–1	1–2	2–3
Delta	2.02	1.61	1.41	0.97	0.91	0.69	1.96	1.62	1.08	1.12	0.68	0.72
Theta	1.52	1.18	1.04	0.69	0.61	0.53	1.46	1.21	0.81	0.79	0.53	0.48
Alpha	1.04	0.77	0.75	0.44	0.46	0.42	0.95	0.76	0.56	0.51	0.38	0.33
Beta	0.59	0.45	0.45	0.28	0.30	0.29	0.57	0.45	0.39	0.32	0.27	0.29
Gamma	0.32	0.27	0.26	0.21	0.20	0.20	0.33	0.31	0.24	0.23	0.20	0.22

On the left lead, for both BSS sessions, the contact pair that was farthest apart (i.e., 0–3) had the highest power differential across all frequency bands. For the contact pairs that were the next farthest apart, 0–2 and 1–3 were remarkably similar, with 1–3 showing a slightly greater average power differential than 0–1. Likewise, for the contact pairs that were closest together, 0–1 and 1–2 were remarkably similar, but 1–2 showed a slightly greater average power differential than 0–1. Both 0–1 and 1–2 were clearly greater than 2–3 in the first session and marginally greater in the second session. Although these findings suggest that contacts 0 and 1 have a stronger LFP signal than contacts 2 and 3, the strongest LFP signal seems to be marginally in closest proximity to contact 1. Contact 0 was determined to be the contact with the greatest clinical benefit and contact 1 was determined to be the contact with the second greatest clinical benefit based on monopolar and bipolar review, see Figure 3; Table 1.

## 4 Discussion

In our case report, we observed that the contact pairs with the highest power across multiple frequency bands included within them the contact with either the greatest clinical efficacy (contact 0 on the right hemisphere) or the second greatest clinical efficacy (contact 1 on the left hemisphere). Our results are more definitive in the right hemisphere than the left hemisphere. This tracks with monopolar and bipolar review yielding more conclusive findings on the right than the left. On the right, the patient showed a half smile, considered to be one of the strongest predictors of clinical response to DBS in patients with OCD (15), only at contact 0. However, on the left, the half-smile occurred at contacts 0 and 1. The patient showed clinical efficacy at contacts 0, 1, and 2 on the left but none at contact 3, which aligns with the LFP data indicating a weaker signal between contacts 2–3 than contacts 0–1 and 1–2. Because the distal end of the lead is implanted with the intention of targeting the area of clinical interest – in this case, the BNST – clinicians may be biased to choose the most distal contact. LFP signals could help clinicians overcome that bias. In the patient, contact 0 was in much closer proximity to the BNST on the right than the left, which may provide a neuroanatomical explanation for why our findings were clearer on the right (see Figure 1). Nevertheless, our results replicated across both LFP recording sessions, separated by 3 months. Overall, our findings suggest that using LFPs when a clear signal is present could increase accuracy in selecting the most clinically efficacious contacts for stimulation, could reduce clinician bias when deciding among contacts that provide seemingly similar clinical benefit, and could potentially reduce the time to clinical benefit for patients with OCD.

Currently, LFP-guided contact selection has been studied exclusively in movement disorders because of readily observable clinical symptoms. Most of these studies have been conducted in Parkinson’s disease because the beta band is a clear neural biomarker that correlates with observable clinical symptoms, namely rigidity (3, 16, 17). Although no other disorders studied have demonstrated such a prominent LFP biomarker as Parkinson’s disease, various LFP signals (e.g., peaks in the beta and theta bands) in other movement disorders, such as essential tremor, dystonia, and epilepsy, have been associated with clinical symptoms and these LFP signals have been shown to normalize with DBS (18). In our study, the strength of the LFP signal across multiple frequency bands corresponded to the clinically optimal contact in the right hemisphere and the second-best contact in the left hemisphere, and a peak in the theta band emerged as a notably strong signal, particularly in the right hemisphere. Another study previously reported on interhemispheric differences in the theta band in OCD (19). When LFP data was recorded in patients with OCD during exposure and response prevention teletherapy, higher theta was associated with lower distress with a medium effect size, but only in the right hemisphere (19). No effect was present in the left hemisphere. Other studies have found the theta band to be meaningfully associated with OCD symptoms (19–21) In an EEG study, patients with OCD had higher theta band EEG coherence in the fronto-occipital region compared to healthy controls (20). In a DBS study targeting the BNST, two patients with OCD exhibited a prominent theta peak when they were shown OCD-provoking images with stimulation turned off (21) When stimulation was turned on, the theta peak was reduced and was similar to the theta peak observed when stimulation was off while they viewed neutral images.

Our study is the first to suggest that LFPs could potentially be used to guide contact selection in patients with OCD. In Parkinson’s disease, beta band LFPs have been used to guide contact selection (1, 5, 8, 10). One study showed that when contacts were selected using LFP signals in the beta band, the clinical efficacy of DBS did not differ from when they were selected using traditional monopolar and bipolar review conducted by highly specialized clinicians (6). Although these advances in Parkinson’s disease are a significant achievement with important implications for clinical care in this population, clinical symptoms in Parkinson’s disease can be modified with DBS in observable ways over a period of seconds to minutes whereas clinical improvements in psychiatric disorders, such as OCD, typically manifest weeks to months after DBS is initiated due to the complex pattern of disordered thoughts and behaviors that underlie OCD (19). Consequently, being able to identify contacts with the greatest likelihood of clinical response using LFP biomarkers would be particularly beneficial in OCD and other disorders with a delayed response to treatment. To date, one case report of a patient with generalized dystonia and a patient with seizures, conditions that typically take days to weeks of DBS before clinical improvement becomes clear, considered how LFPs could inform DBS programming (12). This study, however, did not compare the clinical efficacy of contacts selected with LFPs to traditional monopolar and bipolar review.

Our study is novel but has limitations. Baseline BrainSense Survey LFP data was not available (i.e., from the time after electrodes were implanted but before stimulation was turned on). Future research could be improved by collecting baseline LFPs so that potential neural biomarkers of clinical symptoms could be identified. Furthermore, LFP signals were associated with the contact with greatest clinical benefit for the right hemisphere but the contact with the second greatest clinical benefit for the left hemisphere. On the right, contact 0 was in close proximity to the BNST. Contact 0 was slightly more dorsal on the left relative to the right (see Figure 1). Although this might explain the clearer signal in the right hemisphere, more testing would be needed to determine if this drove our hemispheric differences. Finally, our data is based on a single patient and will need to be validated in a larger patient sample.

## 5 Conclusion

Our findings are the first indication that local field potentials could guide contact selection in patients with OCD. We found that theta band demonstrated the most notable peak, which correlated with the optimal contact in each hemisphere. If validated in a larger sample, this methodology could decrease time to clinical benefit and improve accuracy in patients that are difficult to assess using traditional methods. Further research is needed to determine whether local field potentials could be used to guide finer resolution in programming parameters.

## Data availability statement

The raw data supporting the conclusions of this article will be made available by the authors, without undue reservation.

## Ethics statement

The studies involving humans were approved by University of Colorado COMIRB 14-0554. The studies were conducted in accordance with the local legislation and institutional requirements. The participants provided their written informed consent to participate in this study. Written informed consent was obtained from the individual(s) for the publication of any potentially identifiable images or data included in this article. Written informed consent was obtained from the participant/patient(s) for the publication of this case report.

## Author contributions

KD: Conceptualization, Writing – original draft. EF: Conceptualization, Investigation, Writing – original draft. JTh: Conceptualization, Data curation, Formal analysis, Methodology, Visualization, Writing – review & editing. JTa: Conceptualization, Resources, Visualization, Writing – review & editing. MP: Writing – original draft. SO: Writing – review & editing. RD: Conceptualization, Investigation, Methodology, Project administration, Supervision, Writing – original draft.
