# Supplementary material for: Case report: Clinical efficacy of deep brain stimulation contacts corresponds to local field potential signals in a patient with obsessive-compulsive disorder
Source: Front Psychiatry. 2023 Nov 23;14:1279972. doi: 10.3389/fpsyt.2023.1279972 (PMC10703467; doi:10.3389/fpsyt.2023.1279972)
Supplement: Supplementary file 1 [file Data_Sheet_1.docx]

Supplementary Material

# Supplemental Figures

**Figure 1S.** Diffusor tensor imaging tractography before DBS implantation. We used a deterministic tractography algorithm (Nordic Neurolab) and placed a single seed in the ventral tegmental area [1],[2]. We prefer to be descriptive and state that the tracts seeded from the ventral tegmental area coursed through the anterior limb of the internal capsule (ALIC) despite debate regarding whether the tract representations reflect the medial forebrain bundle (specifically, the superior lateral branch or sIMFB), fibers connecting the subthalamic nucleus to the cingulate [3], or fibers through the ALIC [4].


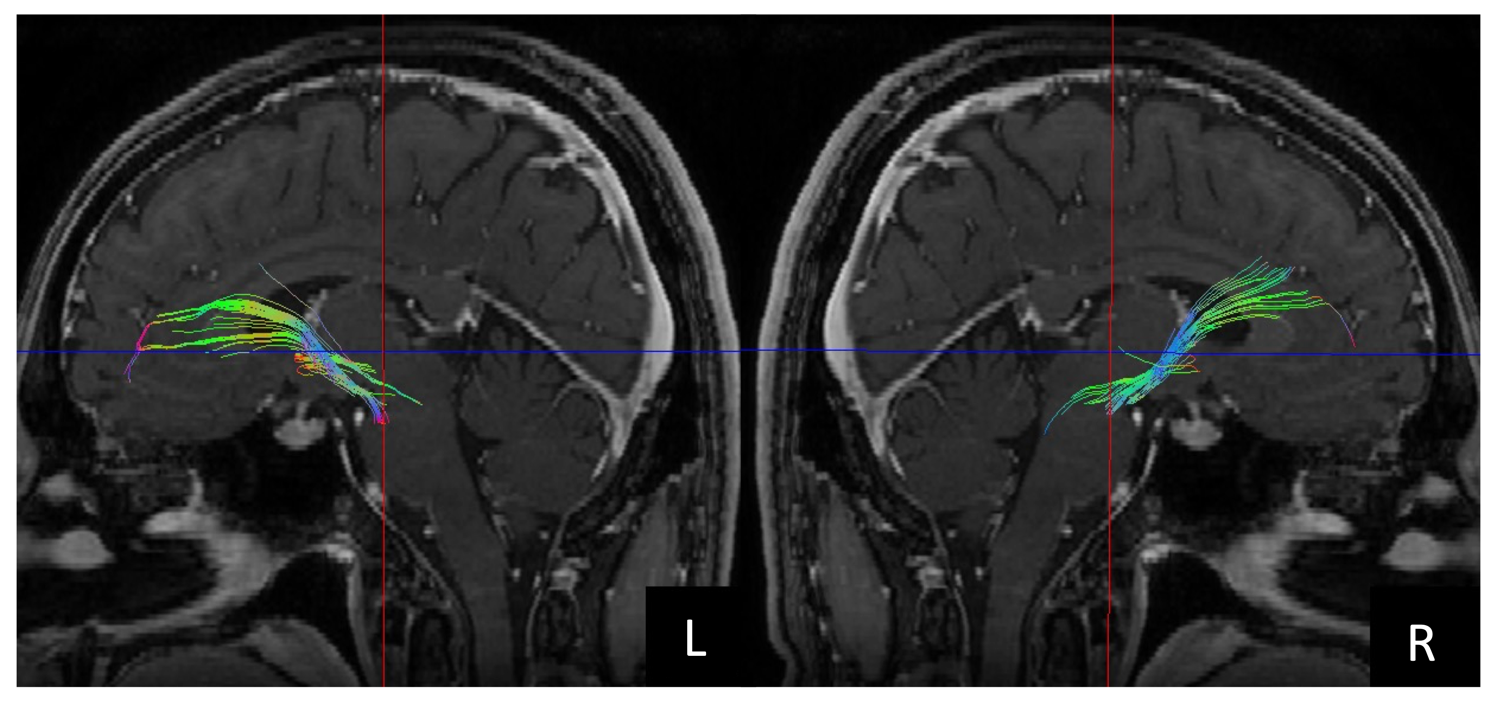


# Supplementary Data

**Patient medication history**

Prior to DBS surgery, the patient’s medications were desvenlafaxine 100mg daily, haloperidol 1mg at night, and memantine 100mg bid. He was also receiving monthly IV ketamine infusions. After DBS surgery, desvenlafaxine and memantine remained the same, and he continued monthly ketamine infusions for 11 months after stimulation was turned on until they were discontinued. Haloperidol was discontinued 5 months after stimulation was turned on due to concern it was contributing to fatigue. Aripiprazole 2.5mg was started 8 months after stimulation was turned on to address an increase in depression symptoms and its use has continued because it has improved mood. When the patient experienced an increase in depression symptoms related to family stressors, Wellbutrin 150mg was started in February of 2023, which occurred after the first BrainSense Survey in January 2023 and before the second BrainSense Survey in April 2023. Although the dose was titrated up to 300mg for one week, the dose was subsequently reduced back to 150mg due to concerns for hypomania.

**Rationale for split stimulation frequencies**

Over a month after stimulation was turned on, the patient’s YBOCS score remained at 20, indicating moderately severe OCD symptoms. As such, we asked the patient if we could test our hypothesis that lowering stimulation frequency on the left could potentially offer additional clinical benefit and the patient was amenable to trying this. When stimulation frequency was decreased from 135 Hz to 60 Hz on the left (while stimulation frequency remained at 135 Hz on the right) and amplitude was increased from 3.5 mAmp to 4.5 mAmp for both hemispheres, the patient described feeling better in the following words: “My mood just went up. I do like this setting. It just feels good.” While it was initially unclear which aspects of the parameter changes (reduced stimulation frequency on the left versus increased amplitude for both hemispheres versus both) played a role in the patient’s improved mood, we inadvertently tested this during a programming session about seven months after his stimulation parameters were changed. During this programming session, the frequency of stimulation for both hemispheres was accidentally set to 135 Hz. The patient remarked that something did not feel right. He later recalled the experience in the following words: “I felt like my thoughts were being dragged down into the depths of despair. I could literally feel my morale sinking. In addition, the freedom and cognitive flexibility I had become accustomed to since changing to the lower frequency stimulation setting began to slip away, and walls of rigid thoughts began forming in my mind.” The clinician then changed the stimulation on the left to 60 Hz and then the patient reported feeling a sense of relief once his optimal therapeutic settings were restored. He later recalled that changing the settings back to his optimal setting was like “imaging being sucked into a vortex of doubt and dread, and suddenly that vortex is gone.”

Three lines of evidence provided the reasoning for our hypothesis that lower stimulation frequency on the left relative to the right could offer clinical benefits in DBS for OCD. First, lower frequency repetitive transcranial magnetic stimulation (0.9 Hz - 1 Hz) has been shown to have inhibitory effects for certain regions of the cerebral cortex in some healthy participants [5]–[7] whereas high frequency stimulation demonstrates excitatory effects [8]. Since it remains unknown whether the effects of DBS are excitatory, inhibitory, or could be either depending on the stimulation parameters and region stimulated, it is plausible that, like TMS, DBS too could have more inhibitory effects at lower stimulation frequencies and more excitatory effects at higher stimulation frequencies. Second, in one clinical population (patients with Parkinson’s disease), when DBS was set to a lower stimulation frequency (60 Hz), certain symptoms improved relative to the higher stimulation frequency typically used (130 Hz) [9]. Third, interhemispheric imbalance is thought to be involved in OCD. Patients with OCD, with their reliance on more habitual behaviors and their impairment in making goal-directed decisions [10], may exhibit dominance of left-brain functions. The left brain is more involved in maintaining habits and directing behaviors with short-term effects whereas the right brain is more important for flexibility of thought, inhibiting immediate or reflexive responses, learning new skills, and considering the broader context and long-term goals [11]. Therefore, differential stimulation of the left versus right hemisphere could be beneficial, especially if it inhibits activity in the left hemisphere to a greater extent than the right hemisphere.

1. Coenen VA, Schlaepfer TE, Goll P, Reinacher PC, Voderholzer U, Tebartz Van Elst L, et al. The medial forebrain bundle as a target for deep brain stimulation for obsessive-compulsive disorder. CNS Spectr. 2017 Jun 1;22(3):282–9.

2. Coenen VA, Panksepp J, Hurwitz TA, Urbach H, Mädler B. Human medial forebrain bundle (MFB) and anterior thalamic radiation (ATR): Imaging of two major subcortical pathways and the dynamic balance of opposite affects in understanding depression. Journal of Neuropsychiatry and Clinical Neuroscience [Internet]. 2012;24(2). Available from: http://neuro.psychiatryonline.org

3. Middlebrooks EH, Domingo RA, Vivas-Buitrago T, Okromelidze L, Tsuboi T, Wong JK, et al. Neuroimaging advances in deep brain stimulation: Review of indications, anatomy, and brain connectomics. American Journal of Neuroradiology. 2020 Sep 1;41(9):1558–68.

4. Haber SN, Yendiki A, Jbabdi S. Four deep brain stimulation targets for obsessive-compulsive disorder: Are they different? Biol Psychiatry. 2021 Nov 15;90(10):667–77.

5. Caparelli EC, Backus W, Telang F, Wang G-J, Maloney T, Goldstein R, et al. Is 1 Hz rTMS always inhibitory in healthy individuals? The Open Neuroimaging Journal. 2012;6:69–74.

6. Chen R, Classen J, Gerloff C, Celnik P, Wassermann EM, Hallett M, et al. Depression of motor cortex excitability by low-frequency transcranial magnetic stimulation. Neurology. 1997;48(5):1398–403.

7. Boroojerdi B, Prager A, Muellbacher W, Cohen LG. Reduction of human visual cortex excitability using 1-Hz transcranial magnetic stimulation. Neurology. 2000;54(7):1529–31.

8. Pascual-Leone A, Valls-Solé J, Wassermann EM, Hallett M. Responses to rapid-rate transcranial magnetic stimulation of the human motor cortex. Brain. 1994;117(4):847–58.

9. Xie T, Vigil J, MacCracken E, Gasparaitis A, Young J, Kang W, et al. Low-frequency stimulation of STN-DBS reduces aspiration and freezing of gait in patients with PD. Neurology. 2015;84:415–20.

10. Gillan CM, Papmeyer M, Morein-Zamir S, Sahakian BJ, Fineberg NA, Robbins TW, et al. Disruption in the balance between goal-directed behavior and habit learning in obsessive-compulsive disorder. American Journal of Psychiatry. 2011;168(7):718–26.

11. McGilchrist I. The Master and His Emissary. Yale University Press; 2009. 576 p.
